# Liver stiffness measured by MR elastography in children and adults with Fontan circulation: defining expected values and clinical associations

**DOI:** 10.1007/s00247-025-06366-4

**Published:** 2025-08-29

**Authors:** Julia Razavi, Andrew T. Trout, Cara E. Morin, Alexander R. Opotowsky, Clayton Smith, Joseph J. Palermo, Julie A. Bonn, Jonathan R. Dillman

**Affiliations:** 1 Department of Radiology, Cincinnati Children’s Hospital Medical Center, University of Cincinnati College of Medicine, 3333 Burnet Avenue, Cincinnati, OH 45229 USA; 2 Department of Pediatrics, Cincinnati Children’s Hospital Medical Center, University of Cincinnati College of Medicine, Cincinnati, OH USA; 3 Heart Institute, Cincinnati Children’s Hospital Medical Center, Cincinnati, OH USA; 4 Division of Gastroenterology, Hepatology, and Nutrition, Cincinnati Children’s Hospital Medical Center, Cincinnati, OH USA

**Keywords:** Adults, Children, Fontan, Liver stiffness, MR elastography

## Abstract

**Background:**

MRI is increasingly used to assess Fontan-associated liver disease (FALD), but the expected range and variability of MR elastography (MRE) shear stiffness measurements as well as their clinical associations remain poorly understood.

**Objective:**

This study aimed to define the range of MR elastography (MRE) measured liver shear stiffness and its clinical associations in a large pediatric and adult cohort of patients post-Fontan.

**Materials and methods:**

This retrospective cross-sectional study included children and adults who underwent baseline clinical liver MRE between February 2013 and June 2024. Mean liver stiffness (kPa) and liver volume (mL) were obtained from clinical reports, while spleen length was measured on coronal T2-weighted images. Clinical data, Fontan conduit size, Fontan pathway pressure, fenestration status, and presence of protein-losing enteropathy were collected within 6 months of imaging. Associations between liver stiffness and clinical variables were assessed using Pearson correlation and multiple linear regression. Clinical characteristics were compared between patients in the lowest (≤ 20th) and highest (≥ 80th) percentiles of liver stiffness.

**Results:**

Our study sample included 206 Fontan patients (mean age 19.7 years; 114 male). Mean liver stiffness was 4.4 ± 1.0 kPa (range 2.1–7.4 kPa). Liver stiffness was associated with aspartate aminotransferase (AST) (*r*=0.23, *P*=0.003), total bilirubin (*r*=0.31, *P*<0.0001), and male sex (4.6 vs. 4.1 kPa; *P*=0.0002), but not with liver volume (*r*=0.06, *P*=0.39) or spleen length (*r*=0.08, *P*=0.25). In a regression model excluding Fontan conduit size and Fontan pressure (*n*=143), male sex (*P* = 0.02), total bilirubin (*P*=0.003), and AST (*P* = 0.048) were independent predictors of liver stiffness (*R*^2^=0.16). In a smaller cohort with available conduit size and pressure data (*n*=32), predictors of liver stiffness were AST (*P*=0.002), ALT (*P*=0.03), Fontan pressure (*P*=0.0004), and male sex (*P* = 0.03) (*R*^2^ = 0.59). Patients with ≥ 80th percentile liver stiffness were more likely to be male (*P*=0.004), had a lower BMI (23.2 vs. 26.3 kg/m^2^, *P*=0.04), and had a higher total bilirubin (1.4 vs. 0.8 mg/dL, *P*=0.01) compared to those with ≤ 20th percentile liver stiffness.

**Conclusion:**

MRE liver stiffness is elevated and highly variable in individuals with Fontan circulation, with higher measurements associated with male sex, liver-related laboratory changes, and Fontan pathway pressure.

**Graphical Abstract:**

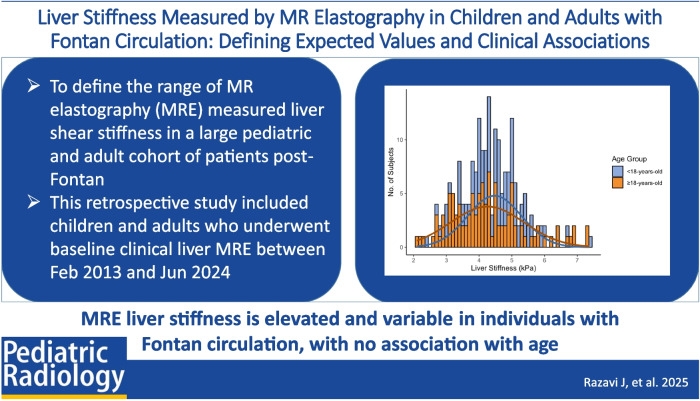

## Introduction

The Fontan procedure, a palliative surgical intervention for patients with functionally single ventricle congenital heart disease, establishes total cavopulmonary anastomosis to redirect systemic venous return directly to the pulmonary arteries, bypassing the heart [1]. While this circulation enables survival into adolescence and adulthood, it is associated with long-term complications, among which Fontan-associated liver disease (FALD) is increasingly recognized as a significant source of morbidity [2, 3].

FALD is characterized by chronic hepatic congestion, progressive fibrosis, portal hypertension, and the development of focal hepatic lesions [3–5]. As the population of patients with Fontan circulation ages, noninvasive methods for routine hepatic surveillance have become essential for early identification and monitoring of liver disease progression [5, 6]. At our institution, our standard surveillance liver MRI protocols for patients post-Fontan include assessments of liver anatomy, spleen length, total liver volume, and liver shear stiffness via magnetic resonance elastography (MRE). Liver stiffness measured by MRE is used as a surrogate marker for the degree of FALD-related liver abnormality, although it currently cannot distinguish between hepatic congestion and fibrosis [7]. Unusually high or progressively increasing liver stiffness may be associated with worse patient outcomes [8, 9], and in our practice, it is seen to warrant further evaluation, which may include liver biopsy to assess for fibrosis or non-FALD causes of liver disease, as well as right-sided cardiac catheterization to measure Fontan pathway pressures and evaluate for outflow obstruction.

Despite the growing clinical emphasis on hepatic monitoring in patients post-Fontan, there remains a paucity of data describing expected values for liver shear stiffness. Furthermore, clinical predictors of increased liver stiffness in this population remain poorly defined. Establishing expected values and identifying relevant clinical correlates are important toward improving detection, risk stratification, and management of FALD.

The purpose of this study is to define the range of MRE-derived liver shear stiffness measurements in a large, mixed pediatric and adult cohort of patients with Fontan circulation. In addition, the study aims to identify clinical variables associated with liver shear stiffness and to evaluate differences in these variables between patients in the lowest (≤ 20th percentile) and highest (≥ 80th percentile) ranges of liver stiffness. By characterizing normative and outlier values and their clinical correlates, our study seeks to enhance understanding of FALD and support the development of risk stratification tools for routine surveillance.

## Methods

This IRB-approved, retrospective study was conducted in a HIPAA-compliant manner with a waiver of documentation of informed consent.

Pediatric and adult Fontan patients who underwent clinical surveillance liver MRI with MRE between February 2013 and June 2024 were identified using an existing Research Electronic Data Capture (REDCap) database that contained all individuals with Fontan circulation. The baseline (first) MRI with MRE was used for patients who had more than one imaging examination during the study period.

**Fig. 1:**
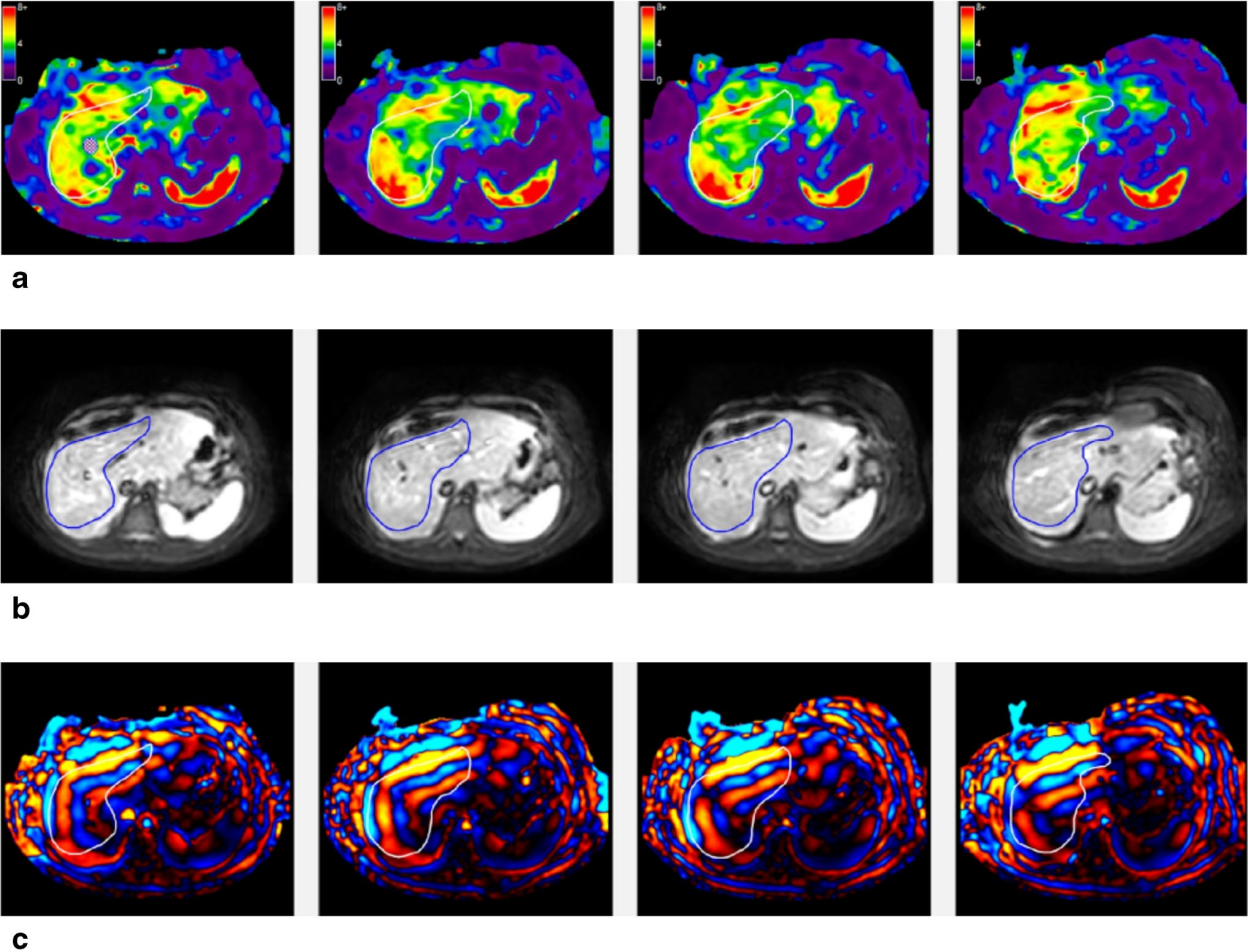
MR elastography performed in a 19-year-old young man with Fontan circulation. **a** MR elastograms are shown at four different anatomic levels through the mid liver. Regions of interest (ROIs) are present on the elastogram images, manually placed by an imaging post-processing laboratory technologist and guided by (**b**) magnitude images and (**c**) wave images. Magnitude images are used to ensure placement of the ROIs within the right lobe and left medial section of the liver, while wave images are used to ensure ROI placement in areas of organized wave propagation. The overall ROI size-weighted mean liver shear stiffness was 4.7 kPa, with individual image stiffness measurements ranging from 4.6 kPa to 4.8 kPa and ROIs ranging from 6234 to 7558 voxels

Mean liver shear stiffness (kPa) and liver volume (mL) were extracted from clinical MRI reports. At our institution, MRE is performed using either a two-dimensional gradient echo or spin echo echo-planar imaging sequence, depending on the clinical protocol in place at the time of examination, and an active driver frequency of 60 Hz. For each examination, four axial images were acquired through the mid-portion of the liver. Mean liver shear stiffness was measured from these images in areas of organized wave propagation, focusing on the right lobe and left medial section of the liver and guided by magnitude images and 95% confidence maps, with values weighted according to the size of each region of interest (ROI) (Fig. 1). All measurements were performed by technologists in our clinical imaging post-processing laboratory. As focal liver lesions in Fontan patients are nearly ubiquitous, commonly multiple, and most often small and benign (e.g., focal nodular hyperplasia-like lesions or regenerative nodules), we do not attempt to avoid such lesions when placing ROIs when performing clinical MRE.

Liver volume was measured offline using an independent workstation by a trained post-processing laboratory technologist as part of our routine clinical workflow, based on segmentation of axial T2-weighted images (Vitrea software; Canon Medical Solutions Corporation, Ōtawara, Tochigi, Japan). Maximum craniocaudal spleen length, which is not routinely clinically reported, was measured by a single investigator using coronal single-shot fast spin echo (SSFSE) images. The spleen was measured from its most superior to most inferior aspects, with oblique measurements allowed. The spleen was not measured in patients with heterotaxy syndrome (i.e., polysplenia or asplenia).

For each participant, clinical and laboratory data were extracted from electronic health records within 6 months of the baseline MRE. Collected variables included sex, age, body mass index (BMI), serum creatinine, total bilirubin, aspartate aminotransferase (AST), alanine aminotransferase (ALT), platelet count, Fontan pressure (measured in mm Hg during cardiac catheterization), Fontan conduit size (mm), Fontan pathway fenestration status (open vs. closed), and history of protein-losing enteropathy (PLE). A diagnosis of PLE was based on a review of clinical diagnoses documented in the electronic health record (e.g., diagnoses listed in problem lists and clinical inpatient and outpatient notes).

### Statistical analysis

Continuous variables were summarized as means and standard deviations, while categorical variables were summarized as counts and percentages. Student’s *t* test was used to compare continuous variables between groups (e.g., patients < 18-years-old vs. ≥ 18-years-old, males vs. females, patients in the lowest [≤ 20th percentile] vs. highest [≥ 80th percentile] ranges of liver stiffness), and Fisher’s exact test was used for categorical variables. Pearson correlation coefficients (*r*) and 95% confidence intervals were calculated to assess associations between continuous variables.

Multiple linear regression analysis, with stepwise variable selection, was performed to identify independent clinical predictors of MRE measured liver shear stiffness. Two different models were created. Our first (base) model included patients that had all of the following clinical variables available: age, sex, BMI, serum creatinine, ALT, AST, platelet count, total bilirubin, fenestration status, PLE, liver volume, and spleen length (*n*=143), with all variables having an opportunity to enter the model. Our second model included patients that had the above base model variables available as well as the Fontan pressure and Fontan conduit size variables (*n*=32). The ANOVA *F*-statistic and its associated significance level were used to assess the overall significance of our regression models and whether the models explain a significant amount of the variation in the dependent variable.

A *P*-value < 0.05 was considered significant for all inference testing. Statistical analyses were performed using MedCalc® Statistical Software version 20.111 (MedCalc Software Ltd; Ostend, Belgium).

## Results

### Study sample

**Fig. 2:**
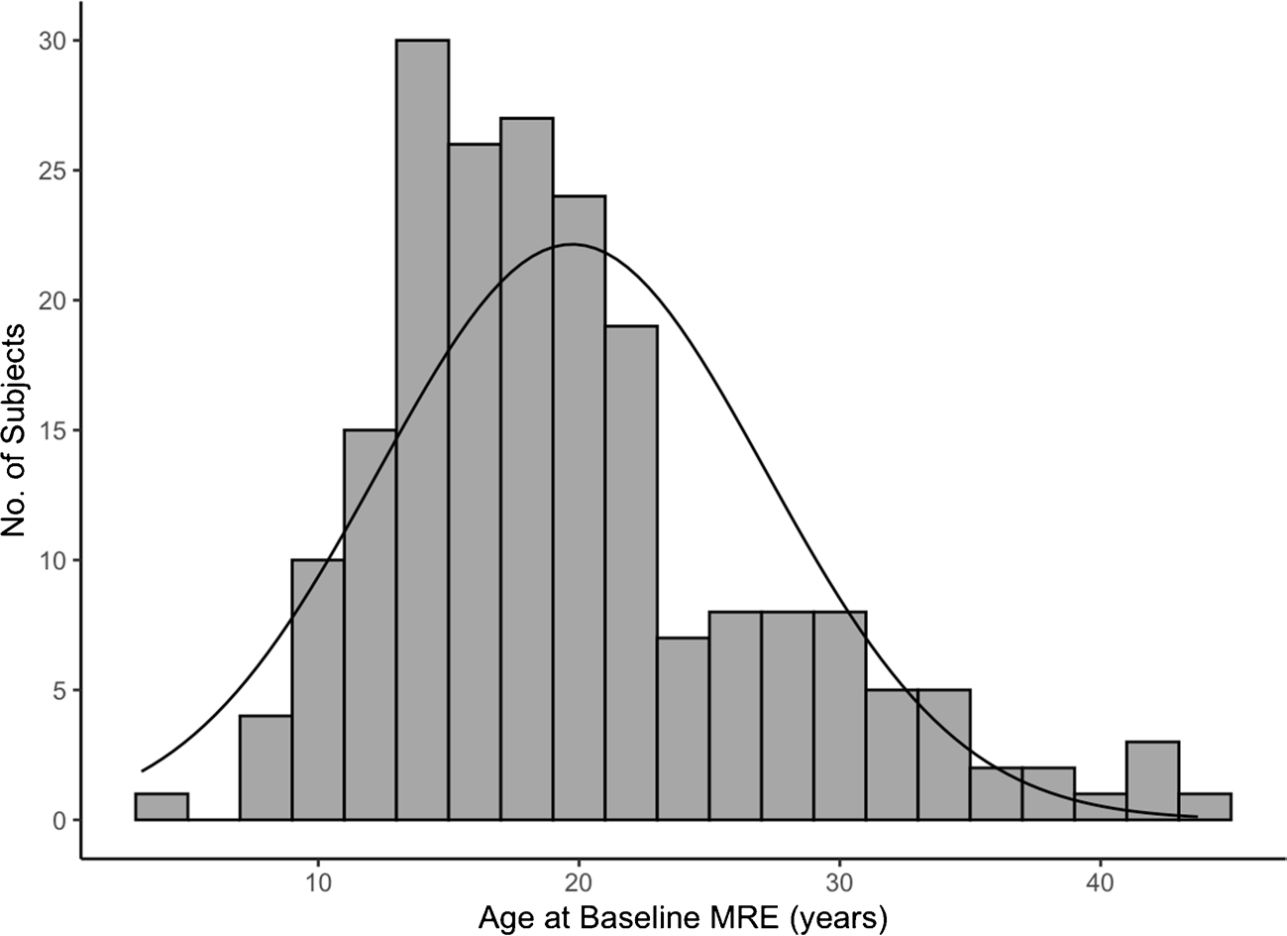
Histogram with overlying normal distribution curve (based on our study sample’s mean age and associated standard deviation) depicting study sample subject age at the time of baseline MR elastography. Mean age was 19.7 years

Our study sample included 206 patients with Fontan circulation who underwent MRE during the study period. Of these, 114 were male (55%) and 92 were female (45%). The mean age of participants was 19.7 ± 7.4 years, with an age range from 3 to 43 years (Fig. 2). Ninety-nine patients were less than 18 years of age (mean age=14.0 ± 2.8 years), while 107 patients were 18 years of age or older (mean age=25.0 ± 6.4 years). The mean time from Fontan operation to baseline MRI was 15.4 ± 6.9 years, with a range from 1 to 35 years. Additional study sample characteristics are presented in Table 1.

**Table 1:** Study sample characteristics presented as means and standard deviations, or counts and percentages

Variable	Mean	Standard deviation	*n*
Age (years)	19.7	7.4	206
Sex	Male – 114 (55.3%)	Female – 92 (44.7%)	206
BMI (kg/m^2^)	23.6	5.9	202
Serum creatinine (mg/dL)	0.7	0.2	170
ALT (U/L)	34.6	18.1	168
AST (U/L)	29.4	14.5	168
Platelets (× 10^9^/L)	200.9	76.3	166
Total bilirubin (mg/dL)	1.1	0.8	163
Fontan pressure (mm Hg)	14.6	3.9	60
Fontan conduit size (mm)	18.4	1.9	116
Protein losing enteropathy	Yes – 18 (8.7%)	No – 188 (91.3%)	206
Fenestration status	Open – 32 (16.2%)	Closed – 166 (83.8%)	198

### Liver stiffness, liver volume, and spleen length

**Fig. 3:**
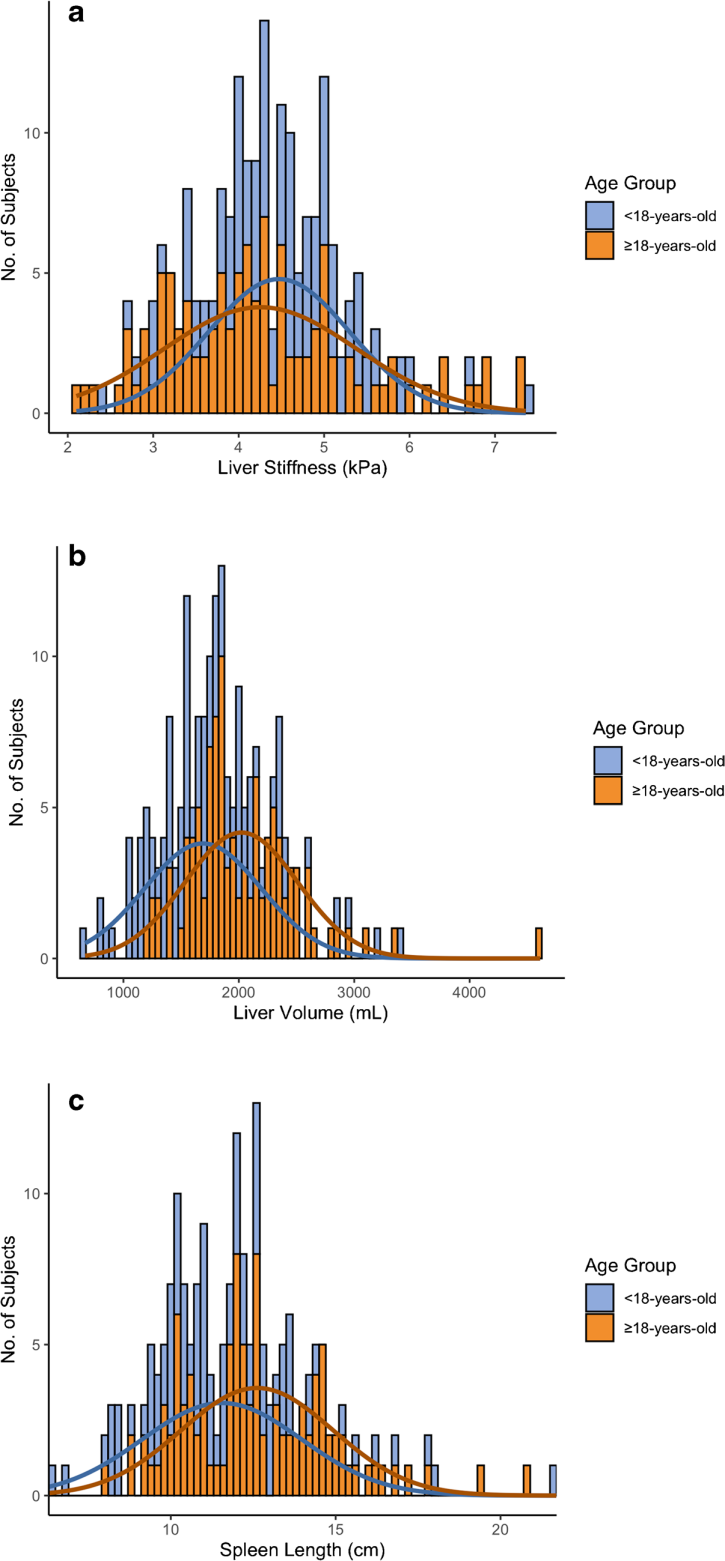
Stacked histograms by age (less than 18-years-old vs. 18-years-old and older) and overlying normal distribution curves (based on the means and standard deviations for these variables from our study sample) depicting (**a**) MR elastography measured liver shear stiffness, (**b**) liver volume, and (**c**) spleen length in our study sample. The length of the spleen was unable to be measured in 10 patients due to heterotaxy syndrome

Liver shear stiffness was highly variable with a mean of 4.4 ± 1.0 kPa, and values ranging from 2.1 kPa to 7.4 kPa. Liver volume also demonstrated substantial inter-individual variability, with a mean of 1860 ± 524 mL and a range extending from 668 to 4611 mL. Spleen length was measurable in 196 individuals with a mean length of 12.1 ± 2.4 cm, ranging from 6.3 cm to 21.7 cm. Spleen length measurements were not available in 10 patients due to coincident heterotaxy syndrome. There was no significant correlation between liver stiffness and either liver volume (*r*=0.06; *P*=0.39) or spleen length (*r*=0.08; *P*=0.25). Histograms of liver stiffness, liver volume, and spleen length are presented by age group in Fig. 3.

**Fig. 4:**
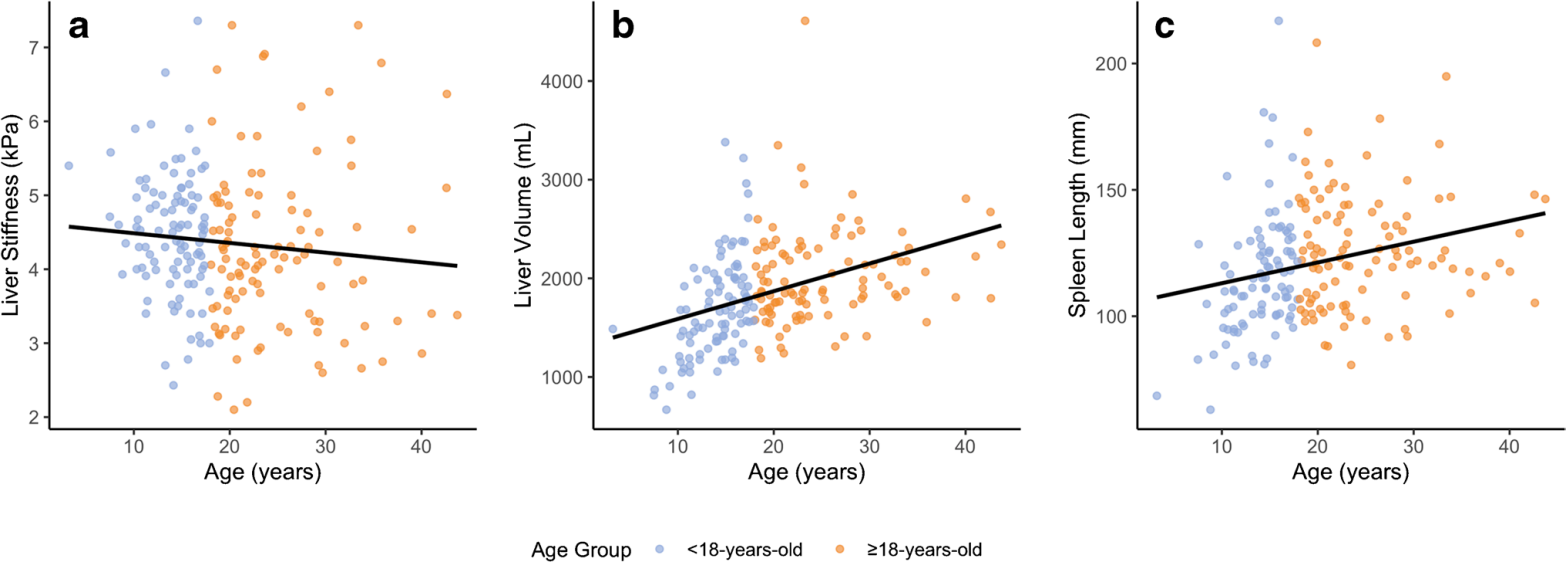
Scatter plots showing the relationships between age and liver shear stiffness, liver volume, and spleen length. There was no significant correlation between age and liver stiffness (*r*=−0.10; *P*=0.16), while there were positive associations between age and both liver volume (*r*=0.40; *P*<0.0001) and spleen length (*r*=0.26; *P*=0.0003)

The relationships between these variables and age were also explored (Fig. 4). There was no significant correlation between liver stiffness and age (*r*=−0.10 [95% CI: −0.23–0.04]; *P*=0.16). There were positive associations between age and both liver volume (*r*=0.40 [95% CI: 0.27–0.51]; *P*<0.0001) and spleen length (*r*=0.26 [95%: 0.12–0.38]; *P*=0.0003).

There was no difference in liver stiffness in Fontan patients 18 years of age or older compared to patients younger than 18 years of age (4.3 ± 1.1 vs. 4.5 ± 0.8 kPa; *P*=0.12). Liver volume (2022.3 ± 487.7 vs. 1688.9 ± 507.7 mL; *P* < 0.0001) and spleen length (12.6 ± 2.3 vs. 11.6 ± 2.3 cm; *P*=0.002) were both larger, on average, in Fontan patients 18 years of age or older compared to younger patients.

### Univariate correlations between liver stiffness and clinical variables

There were significant univariate correlations between liver stiffness and total bilirubin (*r*=0.31; *P*<0.0001) and AST (*r*=0.23; *P*=0.003). Liver stiffness was higher in male than in female patients (4.58 ± 1.0 kPa vs. 4.08 ± 0.9 kPa; *P*=0.0002). No other significant univariate associations were identified (Table 2).

**Table 2:** Univariate correlations between MR elastography measured liver stiffness and clinical variables

Variable	Correlation coefficient (95% confidence interval)	*P*-value	*n*
Age (years)	−0.10 (−0.24 to 0.03)	0.16	206
BMI (kg/m^2^)	−0.12 (−0.26 to 0.01)	0.10	202
Serum creatinine (mg/dL)	−0.02 (−0.17 to 0.13)	0.81	170
ALT (U/L)	0.12 (−0.03 to 0.27)	0.12	168
AST (U/L)	0.23 (0.08 to 0.37)	0.003	168
Platelets (× 10^9^/L)	−0.09 (−0.25 to 0.05)	0.23	166
Total bilirubin (mg/dL)	0.31 (0.15 to 0.43)	< 0.0001	163
Fontan pressure (mmHg)	0.23 (−0.02 to 0.46)	0.08	60
Fontan conduit size (mm)	0.01 (−0.17 to 0.19)	0.88	116

### Multivariable clinical predictors of liver stiffness

In multivariable modeling for the entire sample, three clinical variables were included in our final model for predicting liver stiffness: sex, total bilirubin, and AST (*R*^2^=0.16; *F* ratio = 8.6; *P*<0.0001). Male sex was associated with 0.35 kPa higher stiffness, on average, when compared to female sex (*P*=0.02). A 1 mg/dL increase in total bilirubin was associated with a 0.30 kPa increase in liver stiffness, on average (*P*=0.003), and a 1 U/L increase in AST was associated with a 0.01 kPa increase in liver stiffness, on average (*P*=0.048).

For the smaller subset of patients with available Fontan pathway data (*n*=32), four variables entered our multivariable model, including sex, ALT, AST, and Fontan pressure (*R*^2^ = 0.59; *F* ratio =9.7; *P*=0.0001). Male sex was associated with 0.59 kPa higher stiffness, on average, when compared to female sex (*P*=0.03). A 1 U/L increase in ALT was associated with 0.016 kPa decrease in stiffness, on average (*P*=0.03), while a 1 U/L increase in AST was associated with 0.018 kPa increase in stiffness, on average (*P*=0.002). A 1 mm Hg increase in catheter-measured Fontan pressure was associated with a 0.16 kPa increase in stiffness, on average (*P*=0.0004).

### Differences in clinical variables between patients in the lowest (≤ 20th percentile) versus highest (≥ 80th percentile) ranges of liver stiffness

When comparing clinical variables among subgroups of patients with the lowest and highest ranges of liver stiffness values, patients in the highest stiffness group were more likely to be male (*P*=0.003). BMI was higher in the subgroup with the lowest stiffness compared to the highest stiffness subgroup (26.3 vs. 23.2 kg/m^2^; *P*=0.04), while total bilirubin was higher in the subgroup with highest stiffness compared to the lowest stiffness subgroup (1.4 vs. 0.8 mg/dL; *P*=0.01). There were no significant differences in age, Fontan pressure, Fontan conduit size, or other clinical variables between these two patient subgroups (Table 3).

**Table 3:** Comparisons in clinical variables between patients in the lowest (≤ 20th percentile) versus highest (≥ 80th percentile) ranges of liver stiffness. Data presented as means and standard deviations, or counts and percentages

Variable	≤ 20th percentile	≥ 80th percentile	*P*-value
Age (years)	23.5 (8.3)	19.9 (8.9)	0.054
BMI (kg/m^2^)	26.3 (5.7)	23.2 (7.2)	0.04
Serum creatinine (mg/dL)	0.8 (0.2)	0.7 (0.2)	0.42
ALT (U/L)	33.7 (17.2)	39.5 (22.7)	0.24
AST (U/L)	27.4 (9.3)	35.3 (24.5)	0.09
Platelets (× 10^9^/L)	212.4 (65.4)	196.2 (89.4)	0.42
Total bilirubin (mg/dL)	0.8 (0.5)	1.4 (1.0)	0.01
Fontan pressure (mm Hg)	14.4 (3.9)	17.0 (3.8)	0.10
Fontan conduit size (mm)	18.1 (1.9)	18.3 (2.5)	0.75
Sex	Male – 16 Female – 27	Male – 30 Female – 13	0.004
Protein losing enteropathy	Yes – 2 No – 41	Yes – 5 No – 38	0.43
Fenestration status	Open – 5 Closed – 33	Open – 11 Closed – 30	0.17

## Discussion

In this study, we found that liver stiffness was substantially elevated and highly variable in patients with Fontan circulation compared to published norms for healthy pediatric and adult populations. This finding is consistent with previous studies reporting abnormal liver stiffening in patients post-Fontan, likely reflecting the chronic hepatic congestion and fibrosis associated with Fontan physiology. In healthy children, Trout et al. and Sawh et al. reported mean liver stiffness values of approximately 2.1 kPa and 2.45 kPa, respectively [10, 11]. Trout et al. noted that the 95th percentile of normal liver stiffness was 2.8 kPa, with values unaffected by sex, age, body mass index, MRI scanner vendor, or field strength [10]. Similarly, in healthy adults undergoing liver donor evaluation, Lee et al. reported a mean liver stiffness of approximately 2.1 kPa [12].

Previous studies using magnetic resonance elastography (MRE) have consistently demonstrated elevated liver stiffness in patients with Fontan circulation. Wallihan et al. reported abnormally high liver stiffness in all 14 Fontan patients studied (median age 19 years), with a median value of 4.0 kPa [13]. Similarly, Serai et al. found a mean liver stiffness of 3.9 kPa in a larger cohort of 34 patients (mean age 16.2 years) [7]. In comparison, the mean liver stiffness observed in our study was higher than those previously reported. This difference may be due to our larger and more demographically diverse sample, which included a greater proportion of adults. However, no significant association between liver stiffness and age was identified in either univariate or multivariable analyses, suggesting that other factors beyond age may contribute to the elevated and variable liver stiffness observed.

Our study examined clinical factors associated with increased liver stiffness in patients with Fontan circulation. Male patients consistently exhibited higher liver stiffness on average compared to female patients across both univariate and multivariable analyses. This is noteworthy as other studies measuring liver stiffness in healthy individuals, including in children, have shown no sex-related difference [10]. The exact cause of this association in our study sample is unknown and deserves further investigation, including the possibility of hormonal effects on liver congestion and fibrosis. In multivariable analysis, sex, total bilirubin, and AST were independent predictors of liver stiffness when Fontan pathway factors were not considered. In a secondary multivariable model, limited by a smaller sample size due to data availability, we incorporated catheter-measured Fontan pathway pressure and conduit size (diameter). In this particular model, Fontan pressure, but not conduit size, was significantly associated with liver stiffness, with a 1 mm Hg increase in pressure corresponding to a 0.16 kPa increase in stiffness. Both ALT and AST were also retained in this model but demonstrated divergent associations: increasing ALT was linked to lower liver stiffness, while increasing AST was associated with higher stiffness. This model demonstrated a considerably higher *R*^2^ value than the model for the larger study sample suggesting the potential importance of Fontan pathway pressure as a predictor of liver stiffness, or perhaps more importantly the converse. While serum liver function tests were significant predictors of liver stiffness in both models and in univariate analyses, the clinical relevance of this is uncertain, particularly given opposing trends for ALT and AST, and the likelihood that these are responsive to, rather than drivers of, liver stiffness.

Finally, when comparing liver stiffness in subgroups of patients in the lowest (≤ 20th percentile) versus highest (≥ 80th percentile) liver stiffness, significant differences in BMI and total bilirubin were identified. While elevated total bilirubin was found to be associated with liver stiffness in multiple analyses, and has been shown to increase with progression of other chronic liver diseases, this was the only analysis where BMI was found to be significantly associated with liver stiffness, with a higher BMI on average in patients with lower liver stiffness. The relevance of this relationship is uncertain, although it is possible that patients with a liver stiffness ≥ 80% percentile could be generally sicker with a lower BMI due to frailty or malnutrition.

This study has limitations. Our multivariable model inclusive of Fontan pathway data was constrained by a smaller sample size due to limited availability of catheterization data, which may reduce the robustness and generalizability of findings. Furthermore, this smaller patient group potentially represents a sicker cohort, on average, as these individuals were undergoing an invasive diagnostic procedure to investigate their Fontan pathway. Additionally, the divergent associations of ALT and AST with liver stiffness in the secondary model—one positive and one negative—are of uncertain clinical significance and may reflect underlying variability or model overfitting. Additionally, our study did not assess longitudinal changes in liver stiffness, investigate the relative contributions of hepatic congestion versus fibrosis, or include histologic correlates. Finally, our study used baseline MRI scans rather than the most recent ones in individuals with multiple examinations. It is possible that using follow-up scans could slightly alter our findings, as some patients would be slightly older at the time of the included imaging examination.

In conclusion, liver shear stiffness measured by MRE is significantly elevated in patients after Fontan surgery and exhibits substantial inter-individual variability. Understanding the range and variability of liver stiffness in Fontan patients can support more personalized care and potentially guide diagnostic and management decision-making. Our analyses identified male sex, AST and total bilirubin levels, and higher Fontan pathway pressure (but not conduit size) as predictors of increased stiffness. In contrast, liver volume and spleen length were not significantly associated with stiffness. Despite our efforts, the underlying causes of the significant elevations and wide variation in liver stiffness among Fontan patients remain largely unclear and deserve further study. Our findings underscore the complex nature of hepatic remodeling in patients post-Fontan and highlight the need for further research. Future studies should aim to identify reliable predictors of liver stiffening, monitor longitudinal changes over time, and investigate the relationship between liver stiffness and histologic findings to enhance surveillance and management strategies in this unique patient population.

## Data Availability

Data is available for non-commercial purposes upon reasonable request.
